# Identification of B cell epitopes reactive to human papillomavirus type-16L1- derived peptides

**DOI:** 10.1186/1743-422X-9-199

**Published:** 2012-09-14

**Authors:** Akimasa Fukui, Satoko Matsueda, Kouichiro Kawano, Naotake Tsuda, Nobukazu Komatsu, Shigeki Shichijo, Tetsuro Sasada, Satoshi Hattori, Kimio Ushijima, Kyogo Itoh, Toshiharu Kamura

**Affiliations:** 1 Department of Obstetrics and Gynecology, Kurume University School of Medicine, Kurume, Japan; 2 Department of Immunology and Immunotherapy, Kurume University School of Medicine, Kurume, Japan; 3 Biostatistics Center, Kurume University, Kurume, Japan; 4 Department of Obstetrics and Gynecology, Kurume University School of Medicine, 67 Asahi-machi, Kurume, Fukuoka, 830-0011, Japan

**Keywords:** Human papillomavirus, Prophylactic vaccine, Anti-peptide antibody, Virus-like particles

## Abstract

**Background:**

Persistent infection of human papillomavirus (HPV) types 16 and 18 causes cervical cancer. To better understand immune responses to the prophylactic vaccine, HPV 16/18 L1 virus-like particles (HPV-VLPs), we investigated B cell epitopes of HPV16 L1-derived peptides.

**Methods:**

Sera from mice immunized with HPV-16/18 L1 VLPs were analyzed for their IgG titers against 10 different HPV16 L1-derived peptides (20-mer) that contain human leukocyte antigen (HLA)-class I A-2, A-24 and class II DR.

**Results:**

One 20-mer peptide at positions 300 to 319 was identified as a common B cell epitope in both Balb/c (H-2^d^) and C57BL/6 (H-2^b^) mice. Mapping analysis showed that the 10-amino-acid sequence at positions 304to 313 was an immunogenic portion. It is of note that the binding capability of this 10-mer peptide to the HLA-A2 and HLA-A24 molecules was confirmed by the HLA class I stabilization assay. In addition, one unique 20-mer was determined as a B cell epitope in each strain.

**Conclusions:**

These results might provide new information for better understanding of immune responses to HPV 16 L1.

## Background

Cervical cancer is the second most prevalent cancer in women worldwide. HPV 16 is the most common type associated with cervical cancer [1]. HPV-16/18L1 virus-like particles (HPV-VLPs), which induce neutralizing antibody responses, have been used as a prophylactic vaccine with great success [2,3]. Although the preventive effect of the HPV-VLPs vaccine has been reported to last up to 7.3 years [4], the durability is unclear at the present time, either for the entire vaccinated population or for individuals. This hurdle could be in part overcome if predictable biomarkers were identified. One of the biomarkers could be based on the measurement of specific humoral immune responses to the vaccine. However, little information is presently available with regard to humoral responses against the HPV-VLPs, primarily because of the limited availability of the assay reagents [5,6]. The main aim of this study was to better understand humoral immune responses to HPV16 L1-derived peptides in an animal model.

## Results

### Detection of IgG antibodies in serum of Balb/c mice

**Figure 1:**
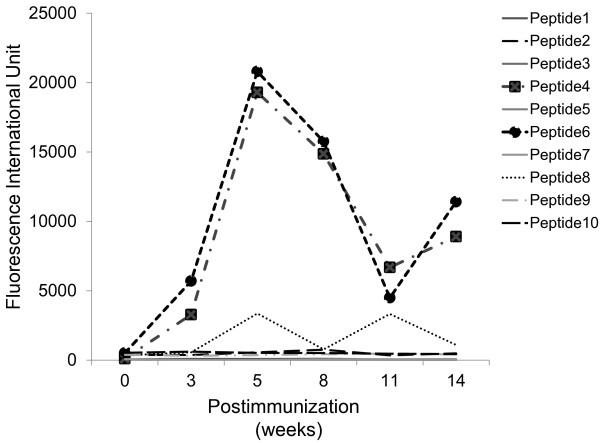
**The kinetics of antibody production in Balb/c mice.** Serum (100-fold dilution) of each of 6 Balb/c mice was obtained before immunization, and 3, 5, 8, 11, 14 weeks after the first immunization followed by the measurement. The representative results are given in fluorescent intensity units (FIU). IgG levels against peptide 4 and peptide 6, but not any others, became detectable at 3 weeks and reached a maximum at 5 weeks, followed by a decline thereafter until 11 weeks (^*^P<0.05; 0 vs. 5 weeks). This analysis was done using Friedman’s test.

Serum of the Balb/c mice was obtained before immunization, and 3, 5, 8, 11, 14 weeks after the first immunization. Each group consisted of 6 mice, and serum from each mouse was independently measured for IgG level. A 100-fold dilution of samples was used to determine the levels of IgG reactive to each of 10 different HPV16 L1-derived peptides (20-mer), and the results were given in fluorescent intensity units (FIU) (Figure 1). Representative results of a kinetic study showed that IgG against peptide 4 and peptide 6, but not any others, became detectable at 3 weeks and reached a maximum at 5 weeks (P<0.05) followed by decline thereafter until 11 weeks. IgG levels were somewhat increased again at 14 weeks, which might be in part a reflection of the second immunization at 3 weeks after the first injection. A dim level of IgG against peptide 8 was detectable at 8 and 11 weeks, but the levels were not significant.

### Detection of IgG antibodies in serum of C57BL/6 N (H-2^b^) mice

**Figure 2:**
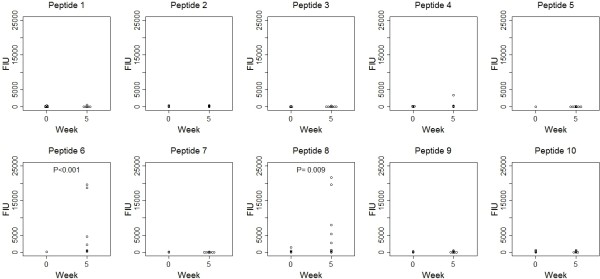
**Antibody production in C57BL/6N mice.** Serum from C57BL/6N mice was obtained before (n = 9)and 5 weeks after the first immunization (n = 9), followed by the measurement. Each of fluorescent intensity units (FIU) from the 9 individual mice was shown. IgG against both peptide 6 and peptide 8, but not any others, was detected at 5 weeks after the immunization. Only the *P* values that were statistically significant(*P* < 0.05)are shown. Wilcoxon rank sum test was used for this analysis.

We then measured IgG levels in serum from the vaccinated C57BL/6N (H-2^b^) mice before and 5 weeks after the immunization to address any difference between Balb/c and C57BL/6N mice. As a result, IgG against both peptide 6 and peptide 8, but not any others, were detected at 5 weeks (P < 0.05) (Figure 2).

### Epitope mapping of peptide

**Figure 3:**
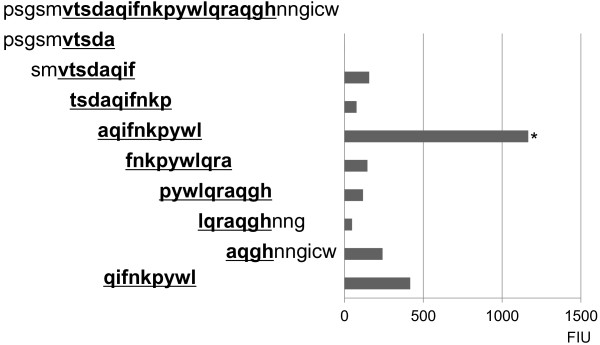
**Epitope mapping of peptide 6 with eight different 10-mer peptides.** Five amino acids sequences from the HPV16 L1-derived sequence at position 295 to 299 were added to the N-terminal of the first 10-mer, and those at position 320 to 324 were added to the C-terminal of the eighth 10-mer (left side of the figure). Each of these eight peptides shares seven amino acids sequences with each other. We also addressed the reactivity to immunized sera of the 9-mer peptide (qifnkpywl) at positions 305 to 313, which had binding motifs to HLA-A2 and HLA-A24. Representative results are given in the right side of the figure. The highest reactivity to one 10-mer (aqifnkpywl at position 304 to 313) is shown (^*^P < 0.05 to any others tested). Wilcoxon rank sum test was used for this analysis.

These results indicated that peptide 6 is the major common B cell epitope shared by the two strains. Subsequently, epitope mapping of peptide 6 was conducted with eight different 10-mers (Figure 3). Five amino acids sequences from the HPV16 L1-derived sequence at position 295 to 299 were added to the N-terminal of the first 10-mer, and those at position 320 to 324 were also added to the C-terminal of the eighth 10-mer. Each of these eight peptides shared seven amino acids sequences with each other. As a result, one 10-mer at amino acid positions of 304–313 of HPV16 L1, aqifnkpywl, was determined to be an immunogenic portion using the antibodies (Figure 3). Then, we addressed the reactivity of the 9-mer peptide (qifnkpywl) at position 305 to 313, which had binding motifs to HLA-A2 and HLA-A24, to immunized sera. A modest level of reactivity to this 9-mer was observed (Figure 3).

### Binding capability of the immunogenic B cell epitope to HLA class I molecules

The identified immunogenic B cell epitope contains binding motifs to the HLA- A2 and HLA-A24 molecules. Therefore, we examined whether they actually bind to the HLA-A2- and HLA-A24 molecules by the HLA class I stabilization assay with TAP-deficient cell lines RMA-S/A2 and RMA-S/A24. As illustrated in Table 1, both of the 9-mer (qifnkpywl) and 10-mer (aqifnkpywl) peptides showed substantial binding capability to HLA-A2 and HLA-A24.

**Table 1:** HLA-binding capability of the B cell epitopse derived from HPV16 L1

**Peptide**	**Sequence**	Binding capability (%)*	
		**HLA-A2**	**HLA-A24**
9-mer	QIFNKPYWL	66.8	40.3
10-mer	AQIFNKPYWL	74.9	51.7
Flu M1	GILGFVFTL	116.7	ND
EBV	TYGPVFMCL	ND	170.6
K-Ras	KLVVVG AGGV	3.3	3.8

*HLA-A2 or HLA-A24-binding capability was estimated by increase in mean fluorescence intensity (MFI), determined by flowcytometry after staining of RMA-S/A2 or RMA-S/A24 cells with anti-HLA-A2 or anti-HLA-A24 mAb, as follows; MFI increase (%) = (MFI with a given peptide – MFI without peptide)/(MFI without peptide) X 100. As positive controls, an HLA-A2-binding peptide derived from influenza virus M1 (Flu M1) or an HLA-A24-binding peptide derived from Epstein-Barr virus LMP2 (EBV) was used. As a negative control, a peptide derived from oncogene K-ras was used. ND, not determined.

## Discussion

We analyzed immune responses to the 10 different HPV-VLP L1-derived peptides (20-mers) that had binding motifs to both HLA-class I (A2 or A24) and HLA-class II (DR) in animal model. We used BALB/c and C57BL/6N mice that have been regarded as Th2- and Th1-skewed strains, respectively, and widely known to express different immune responses in normal and pathological states [7]. When we examined humoral immune responses to the 10 different HPV-VLP L1-derived peptides in the Th2-skewed BALB/c mice, the levels of IgG to the peptide 4 and peptide 6 were clearly elevated in the sera after immunization with HPV-VLPs. We also used the Th1-skewed C57BL/6N mice to examine whether the selection of mouse strains tested has considerable and variable impacts on humoral immune responses to HPV-VLPs. In the C57BL/6N mice, one of the identified peptides , peptide 6 , but not another one (peptide 4), was also immunogenic, suggesting that the peptide 6 is a major common B cell epitope peptide in mice. Notably, our preliminary study has shown that the peptide 6 is also immunogenic in humans who are vaccinated with HPV-VLPs (data not shown), suggesting that the peptide 6 is a common immunogenic B cell epitope shared between mice and humans. A dim level of IgG against peptide 8 was detected in Balb/c mice; thus, peptide 8 might also contain a common epitopic portion. This issue remains to be studied further.

Mapping analysis of peptide 6 showed 10-mer (aqifnkpywl) at positions of 304 to 313 of HPV16 L1 as an immunogenic portion. In addition, the 9-mer peptide (qifnkpywl) at position 305 to 313 with binding motifs to HLA-A2 and -A24 molecules was also recognized by immunized sera. It is of note that both these 9-mer and 10-mer peptides showed substantial binding capability to HLA-A2 and HLA-A24 molecules, the two dominant HLA-class I A types among Japanese and other ethnics [8]. The peptide 6 at positions 300 to 319 of HPV16 L1 also contains binding motifs to HLA-class-II DR 1, 3, and 4 molecules, the dominant molecules expressed in Japanese and other ethnics [9]. Therefore, the peptide 6 and its fragment might be one of the appropriate candidate antigenic sequences for monitoring immune responses to HPV-VLP after vaccination both in animal model and humans. This hypothesis is now under investigation in humans.

Whether the vaccine-induced IgG to the peptide 6 or 8 possesses biological activity to either neutralize HPV infection or facilitate the prophylactic effect of the HPV 16/18L1 vaccine needs to be studied in near future. In addition, since we have known that the same epitopes are often recognized by both B cells and T cells [10], T cell responses to the peptide 6 and its fragment may also be of great interest.

## Conclusions

In summary, one 20-mer peptide at positions 300 to 319 was identified as a common B cell epitope in both Balb/c (H-2^d^) and C57BL/6N (H-2^b^) mice. These results might provide new information for better understanding of immune responses to HPV 16L1.

## Materials and methods

### Immunization of mice

Female Balb/c (H-2^d^) and C57BL/6N (H-2^b^) mice used in this study were 6 weeks of age and were maintained in a pathogen-free environment. Balb/c mice (H-2^d^) were mainly provided for the study because they have been regarded as a Th2- skewed strain [7] and the binding motifs of the peptides to the H-2^d^ class I-A molecules were similar to those of human leukocyte antigen (HLA) class I-A2402, which are expressed in 60% of Japanese [11]. We administered the bivalent HPV-16/18 virus-like particle AS04 vaccine, containing 2 μg each of HPV-16 and HPV-18L1 VLPs with AS04 adjuvant that contained 50 μg aluminum hydroxide and 5 μg 3-deacylated monophosphoryl lipid A. Mice were immunized twice (day 1 and day 21) into the muscle at the thigh with HPV16/18 VLP AS04 vaccine based on the report by Didierlaurent et al. [12].

All experiments with live animals were performed under the institutional guidelines of the animal experiment in Kurume University after the approval by the Committee of Animal Experiment in Kurume University (Approval Number: 2010-107-1).

### Collection of serum samples

The blood of each mouse was collected from the orbital sinus. Blood collection was performed before vaccination, and 3, 5, 8, 11, and 14 weeks after the first immunization in Balb/C (n = 6 in each group). In C57BL/6 mice, blood was collected before (n = 9) and 5 weeks after the first immunization (n = 9). Collected blood was allowed to coagulate by keeping it at room temperature for 1 h followed by centrifugation and collection. Serum from each mouse was sealed and stored at −80°C until use. Measurement of IgG titers was conducted simultaneously to avoid possible *in vitro* biases.

### Peptides

Ten different HPV16 L1-derived peptides (20-mer) with binding motifs to both HLA-class I (A2 or A24) and HLA-class II (DR) were selected by the web software (MULTIPRED) (Table 2). This choice was based on consideration of future applications to the human immune system. For epitope mapping, 8 different 10-mer and one 9-mer peptides were selected from the 20-mer peptide 6. These peptides were purchased from Greiner Bio-One (Thermo Fisher Scientific, Ulm, Germany). Each peptide was dissolved in dimethyl sulfoxide (DMSO), stored at −80°C.

**Table 2:** HPV16 L1-derived peptides used in this study and their binding motifs to HLA-A2 and -A24

	**DR**		**A2**			**4A2**		
	**position**	**Amino acid sequence**	**position**	**Amino acid sequence**	**Score**	**position**	**Amino acid sequence**	**Score**
Peptide 1	54-73	KPNNNKILVPKVSGLQYRVF	60-68	I**L**VPKVSG**L**	30	59-68	K**I**LVPKVS**G**L	14
Peptide 2	392-422	HSMNSTILEDWNFGLQPPPGG	398-406	I**L**EDWNFG**L**	23	397-406	**TI**LEDWNF**G**L	16
Peptide 3	62-81	VPKVSGLQYRVFRIHLPDPN	67-75	G**L**QYRVFR**I**	22	66-75	S**G**LQYRVF**R**I	24
Peptide 4	112-131	PLGVGISGHPLLNKLDDTEN	118-126	S**G**HPLLNK**L**	22	117-126	I**S**GHPLLN**K**L	12
Peptide 5	243-262	GDSLFFYLRREQMFVRHLFN	249-257	Y**L**RREQMF**V**	22	248-257	F**Y**LRREQM**F**V	12
Peptide 6	300-319	VTSDAQIFNKPYWLQRAQGH	305-313	Q**I**FNKPYW**L**	21	305-313	Q**I**FNKPYW**L**	12
Peptide 7	144-162	RECISMDYKQTQLCLIGCK	148-156	S**M**DYKQTQ**L**	20	148-156	S**M**DYKQTQ**L**	11
Peptide 8	293-312	PTPSGSMVTSDAQIFNKPYW	298-306	S**M**VTSDAQI	20	298-306	S**M**VTSDAQ**I**	10
Peptide 9	384-403	TADVMTYIHSMNSTILEDWN	390-399	Y**I**HSMNST**I**L	20	389-398	T**Y**IHSMNS**T**I	23
Peptide 10	152-171	KQTQLCLIGCKPPIGEHWG	157-165	C**L**IGCKPP**I**	23	156-165	L**C**LIGCKP**P**I	12

Anchor residues for HLA class I are shown in boldface.

### Preparation of xMAP beads

The xMAP carboxylate beads and Luminex system platform were obtained from Luminex Corp. (Austin, TX) as reported previously [13]. The 96-well filter plates (MABVN12) and vacuum manifold apparatus (MAVM 09601) were from Millipore Corp. (Bedford, MA). Biotinylated goat anti mouse IgG (gamma chain-specific) (SouthernBiotech, AL) was purchased from Vector Laboratories Inc. (Burlingame, CA). Streptavidin-PE (S-866) was purchased from Molecular Probes (Eugene, OR). 1-Ethyl-3-[3-dimethylaminopropyl] carbodiimide hydrochloride (EDC, 22980) was obtained from PIERCE (Rockford, IL). Peptides were coupled to xMAP beads according to the modified manufacturer’s instructions as reported previously [13]. In brief, 100 μ of xMAP beads were washed with 0.1 M MES buffer, pH 7.0, followed by mixing with 100 μl of peptide (1 mg/ml in 0.1 M MES buffer, pH 7.0). The peptide-loaded beads were incubated with EDC (1 mg/ml) at room temperature for 30 min in darkness, and then incubated twice more under the same conditions, after which the beads were washed with 0.05% Tween 20-PBS. Finally, the beads were treated with 2-aminoethanol for 15 min at room temperature in darkness, then washed twice and re-suspended with 1 ml of 0.05% NaN_3_ in Block-Ace.

### Anti-peptide antibody measurement by multiplexed bead-based Luminex assay

Blood samples were obtained from each of the mice at each scheduled point. Peptide-specific IgG levels in serum were measured by flowmetry assay using the Luminex system as reported previously [13]. In brief, serum was incubated with 100 μl of the peptide-coded beads for 1.5 hours at room temperature in a 96-well filter plate on a plate shaker. After incubation, the plate was washed using a vacuum manifold apparatus and incubated with 100 μl of biotinylated goat anti mouse IgG (gamma chain-specific) for 1 hour at room temperature on a plate shaker. The plate was then washed, 100 μl of streptavidin-PE was added to the wells, and the plate was incubated for 40 min at room temperature on a plate shaker. The bound beads were washed three times followed by the addition of 100 μl of Tween 20-PBS into each well, and the plate was placed for 3 min on a plate shaker.

### HLA class I stabilization assay

The actual binding of the peptides to HLA-A2 or HLA-A24 molecules was evaluated by MHC class I stabilization assay with the TAP2-deficient RMA-S cells stably transfected with the HLA-A*0201 gene (RMA-S/A2) or with the HLA-A*2402/K^b^ gene (RMA-S/A24), according to a previously reported method with several modifications [14]. Briefly, RMA-S/A2 or RMA-S/A24 cells (5x10^5^ cells per well in a 24-well plate) were cultured for 18 hours at 26°C in 1 ml of RPMI 1640 medium (Invitrogen Inc, Carlsbad, CA) containing 10% FBS (MP Biologicals, Solon, OH) in the presence of synthetic peptides (25 μg/ml) and β2-microglobulin (2 μg/ml; Fitzgerald Industries International, Acton, MA). After washing, the cells were cultured for 3 hours at 37°C, and then stained with anti-HLA-A2 mAb (BB7.2; BD Bioscience, San Jose, CA) or anti-HLA-A24 mAb (One Lambda, Inc. Canoga Park, CA), followed by incubation with PE-conjugated rabbit anti-mouse IgG Ab (MP Biomedicals, Solon, OH). After washing, the cells were suspended with 1 ml of PBS containing 1% formaldehyde, and analyzed with FACSCanto (BD Bioscience). The binding capability of each peptide to HLA-A2 or HLA-A24 molecules was evaluated by the increase in mean fluorescence intensity (MFI) assessed by flow cytometry, as follows: MFI increase (%) = (MFI with a given peptide – MFI without peptide)/(MFI without peptide) X 100. As positive controls, an HLA-A2-binding peptide derived from influenza virus M1 (Flu M1, GILGFVFTL) or an HLA-A24-binding peptide derived from Epstein-Barr virus LMP2 (EBV, TYGPVFMCL) was used. As a negative control, a peptide derived from oncogene K-ras (KLVVVG AGGV) was used.

### Statistics

The statistical significance of the data was determined using Friedman`s test and the Wilcoxon rank sum test. P-values less than 0.05 were considered statistically significant.

## Abbreviations

HPV: Human papillomavirus; VLPs: Virus-like particles; HLA: Human leukocyte antigen; FIU: Fluorescent intensity units.

## Competing interests

The authors declare that they have no competing interests.

## Authors’ contributions

A.F. performed most of the experiments and involved in manuscript preparation. T.S. and N.K. coordinated laboratory manipulation. KK proposed the hypothesis of this study edited the manuscript. K.K., N.T., K.U., K.I. and T.K. designed this study. S.S. and A.F. designed the peptides analyzed. A.F., S.M. gave vaccination to mice and obtained blood sample, A.F., S.M., N.K. and T.S. measured the antibody. A.F., T.S., S. H., and K.I. analyzed the data. KI and TK are the project leaders and were involved in project design, manipulation, data analysis and finalization of the manuscript. All authors read and approved the final manuscript.
